# A Novel LC-MS/MS Method for the Measurement of Elexacaftor, Tezacaftor and Ivacaftor in Plasma, Dried Plasma Spot (DPS) and Whole Blood in Volumetric Absorptive Microsampling (VAMS) Devices

**DOI:** 10.3390/pharmaceutics17020200

**Published:** 2025-02-06

**Authors:** Federica Pigliasco, Alessia Cafaro, Sebastiano Barco, Federico Cresta, Rosaria Casciaro, Nicoletta Pedemonte, Francesca Mattioli, Carlo Castellani, Giuliana Cangemi

**Affiliations:** 1 Unit of Biochemistry, Pharmacology and Newborn Screening, Central Laboratory of Analysis, IRCCS Istituto Giannina Gaslini, 16147 Genoa, Italy; federicapigliasco@gaslini.org (F.P.); alessiacafaro@gaslini.org (A.C.); giulianacangemi@gaslini.org (G.C.); 2 Cystic Fibrosis Center, IRCCS Istituto Giannina Gaslini, 16147 Genoa, Italyrosariacasciaro@gaslini.org (R.C.);; 3 UOC Genetica Medica, IRCCS Istituto Giannina Gaslini, 16147 Genoa, Italy; 4 Pharmacology & Toxicology Unit, Department of Internal Medicine, University of Genoa, Viale Benedetto XV 2, 16132 Genoa, Italy; 5 Clinical Pharmacology Unit, EO Ospedali Galliera, Mura Delle Cappuccine, 14, 16128 Genoa, Italy

**Keywords:** CFTR modulators, cystic fibrosis, liquid chromatograph–tandem mass spectrometry, microsampling, pediatric pharmacology

## Abstract

**Background:** The combination of ivacaftor, tezacaftor and elexacaftor (ETI) is approved for patients with cystic fibrosis (CF) aged two years and older and at least one F508del mutation in the CFTR gene. Variability in ETI treatment response has been repeatedly reported, and its reasons are unclear and understudied. **Objectives:** We present a novel liquid chromatography–tandem mass spectrometry (LC–MS/MS) method for the rapid and simultaneous quantification of ETI in plasma, dried plasma spots (DPS), and whole blood volumetric absorptive microsampling (VAMS). **Methods:** The method utilizes a rapid extraction protocol with 200 μL methanol after the addition of deuterated internal standards. Chromatographic separation was achieved using a reversed-phase Hypersil Gold aQ column (Thermo Fisher Scientific). The method was validated according to ICH (International Council on Harmonisation) guidelines M10 for bioanalytical method validation, demonstrating linearity in the concentration range 0.020–12.000 µg/mL. It was also proved accurate and reproducible with no matrix effect. This method was applied to anonymized samples from patients undergoing ETI treatment: eight plasma and DPS and five VAMS samples were analyzed. **Results:** ETI concentrations measured in plasma and DPS were interchangeable, whereas ETI concentrations in VAMS were lower than in plasma, as expected for molecules with high plasma protein binding (99%). A correction factor based on the hematocrit value was used to calculate the equivalent plasma concentration from VAMS concentrations. **Conclusions:** This method is suitable for pharmacokinetic (PK) studies and could facilitate the centralization of samples to specialized laboratories, supporting multicenter studies.

## 1. Introduction

Cystic fibrosis (CF) is an autosomal recessive disease caused by mutations in the cystic fibrosis transmembrane conductance regulator (CFTR) gene, a channel protein responsible for regulating ion transport across cell membranes [1]. CFTR modulators are a novel class of drugs designed to target CFTR protein with the aim of restoring its defective function in patients affected by CF [2]. The development and use of CFTR modulators represent a transformative therapeutic approach to the treatment of CF [2,3,4,5]. Kaftrio^®^ is a combination of three pharmacological agents, elexacaftor (ELX), tezacaftor (TEZ), and ivacaftor (IVA) (ETI), and is approved for patients aged two years and older with at least one F508del mutation in the *CFTR* [6]. Currently, Kaftrio^®^ is prescribed in a standard fixed dose for adults and adjusted by weight in children. In patients undergoing ETI treatment, variability in treatment response, including those with good adherence to treatment, has been observed [2,7]. This phenomenon may have several, possibly complementary, explanations. It has been shown that the presence of additional variants in cis with the F508del mutation may influence or even negate clinical response to ETI [2]. Given the significant impact of CF-related pathological changes on drug absorption, distribution, metabolism, and elimination (ADME), pharmacokinetic (PK) between-subject variability (BSV) is expected [2,4]. Patients with CF undergo a heavy treatment burden, which in turn implies a high probability of drug–drug interactions (DDIs) with ETI. This is mainly due to the metabolism of ETI in the liver, which is predominantly mediated by cytochrome P450 (CYP450) enzymes, in particular CYP3A4 and CYP3A5A [2,8,9]. A definitive correlation between PK parameters and ETI efficacy has not yet been established and the optimal timing for Therapeutic Drug Monitoring (TDM) sampling remains undetermined [2,10]. A significant correlation between the minimum concentration (C_min_) measured in pre-administration ETI samples and key efficacy parameters such as sweat chloride test (Cl-), forced expiratory volume in the first second (FEV1%) and body mass index (BMI) has not been established [10,11]. Additional studies are essential to comprehensively investigate the role of PK in the variability of treatment response to ETI [2,8,10]. Currently, the availability of bioanalytical methods capable of simultaneously quantifying ETI in biological samples is limited to a few centers worldwide [10,12,13,14,15,16,17]. A crucial factor contributing to such scarcity is the lack of commercially available In Vitro Diagnostic Regulation (IVDR)-certified kits for ETI, and the consequent need for laboratories to develop their own in-house assays [10]. However, developing such methods in-house requires specialized personnel with expertise in LC-MS/MS method development, validation, optimization, and troubleshooting, which is particularly challenging and resource-intensive for many laboratories. Microsampling, an innovative sampling technique, has gained prominence as a minimally invasive method for collecting small volumes of biological fluids. When coupled with liquid chromatography–tandem mass spectrometry (LC-MS/MS), microsampling enables precise and accurate quantification of drug concentrations in biological matrices [18]. This approach presents a valuable opportunity to explore the pharmacological profile of ETI with enhanced sensitivity and reduced patient burden. Moreover, it could facilitate access to this assay for patients in centers that lack the capability to perform it. Volumetric Absorptive Microsampling (VAMS) represents an innovative technique that enables the collection of a precise volume of whole blood microsampling [19,20,21]. Dried Plasma Spot (DPS) is a technique that involves spotting fixed-volume plasma microsampling onto traditional cellulose paper substrates [18]. The aim of this paper is to describe the first LC-MS/MS method for the quantitation of ETI in plasma, DPS and VAMS. This advancement holds significant promise in improving our understanding of ETI PK variability. Furthermore, it enables the centralization of samples to a specialized laboratory, facilitating multicenter studies [22].

## 2. Materials and Methods

### 2.1. Chemicals and Reagents

Elexacaftor (ref. E201545), Tezacaftor (ref. T321510) and Ivacaftor (ref. I940600) were obtained from Toronto Research Chemicals (North York, ON, Canada). Elexacaftor d-3 (E201547), Tezacaftor d-4 (T321512) and Ivacaftor d-9 (ref. I940603) were purchased from Spectra 2000 (Rome, Italy). LC–MS/MS-grade formic acid (ref. 607001000), LC–MS/MS-grade acetonitrile (ref. 1.00029) and dimethyl sulfoxide (DMSO) were obtained from Sigma (Milan, Italy); LC–MS/MS-grade methanol (ref. 414942) was obtained from Carlo Erba reagents (Cornaredo, Milan, Italy). All reagents have 98% purity. All solutions were prepared using HPLC-grade water from a Milli-Q Plus water purification system. HPLC mobile phases were filtered through Millipore membrane filters [0.45 µm] (Millipore, Vimodrone MI, Italy).

### 2.2. Calibration Curve, Quality Control, Stock and Working Solutions Preparation

ELX, TEZ and IVA were dissolved in DMSO to prepare stock solutions at concentrations of 2.600 mg/mL, 1.600 mg/mL, and 2.500 mg/mL, respectively. Stock solution of internal standards (IS) of Tezacaftor d-9 (TEX d-9) (0.5 mg/mL), Elexacaftor d-3 (ELX d-3) (0.500 mg/mL) and Ivacaftor d-9 (IVA d-9) (1 mg/mL) were prepared by dissolving any substance in DMSO. A working solution of ELX, TEZ and IVA (100 µg/mL) was obtained by diluting the respective stock solutions in methanol. An IS mixture working solution containing ELX d-3, TEX d-9 and IVA d-5 was obtained by diluting each stock solution to achieve a final concentration of 0.500 µg/mL for each IS. Calibrators and Quality Controls (QCs) were generated by diluting the working solutions in blank plasma collected from healthy adult volunteers. An 8-point calibration curve for ELX, TEZ and IVA was established with concentrations ranging from 0.020 to 12.000 µg/mL, specifically: 0.020 (lower limit of quantification, LLOQ), 0.050, 0.120, 0.30, 0.770, 1.920, 4.800 and 12.000 µg/mL. Additionally, three QCs were prepared at concentrations of 0.060 µg/mL (QC low), 0.400 µg/mL (QC medium) and 10.000 µg/mL (QC high).

### 2.3. Human Samples

We evaluated the applicability of the described bioanalytical method based on LC-MS/MS for the measurement of ETI in plasma, DPS and whole-blood VAMS. The method followed the ethical standards of the Institutional and National Research Committee and the 1975 Helsinki Declaration, revised in 2013. Written informed consent for collecting residual samples and anonymized clinical data was obtained from all patients or their legal representatives according to the privacy policy of the IRCCS Istituto Giannina Gaslini, Genoa, Italy. In accordance with international guidelines on bioanalytical method validation, we analyzed samples from eight patients treated with Kaftrio^®^ and followed them at IRCC Istituto Giannina Gaslini. Leftover whole blood samples for routine analysis with ethylenediaminetetraacetic acid (EDTA) as an anticoagulant were centrifuged at 20,000× *g* for 5 min at 4 °C to obtain plasma. Plasma and DPS samples were obtained from all of them while VAMS were obtained from five of them. All samples were obtained before the ETI morning administration.

### 2.4. Sample Pre-Treatment

#### 2.4.1. Extraction from Plasma

A 50 μL aliquot of plasma sample (calibrators, QC or patient’s) was mixed with 200 μL aliquot of methanol (MeOH) following the addition of 5 µL of IS mixture working solution (0.5 μg/mL). After thorough vortexing, the samples were centrifuged at 20,000× *g* for 5 min at 4 °C and the supernatants were then transferred to autosampler glass vials for injection into the LC-MS/MS system.

#### 2.4.2. Extraction from DPS

Calibration standards, QCs and patient samples (20 μL each) were carefully applied to Ahlstrom 226 filter paper using a calibrated pipette and allowed to air dry at room temperature (25 ± 2 °C) for 1 h. Each DPS was then punched to obtain eight disks, each with a diameter of 3.2 mm (approximately 3.3–3.4 μL of plasma). These disks were placed into 1.5 mL Eppendorf tubes and extracted with 200 μL of methanol, with the addition of 5 μL of IS mixture working solution (0.500 μg/mL). After a 10 min incubation at 37 ± 1 °C, the samples were centrifuged at 20,000× *g* for 5 min at 4 °C. The supernatants were then collected into autosampler glass vials for injection into the LC-MS/MS system.

#### 2.4.3. Preparation and Extraction from VAMS

VAMS (MITRA^®^, Neoteryx, Torrance, CA, USA) samples were prepared using the 30 µL device model following the manufacturer’s instructions. The upper part of the tip was carefully dipped into a volume of blood, ensuring that the tip was not fully immersed to avoid overfilling. Once the tip turned completely red, it was held in place for an additional 2 s. The devices were air-dried for 1 h at room temperature and protected from light. VAMS tips were placed into 1.5 mL Eppendorf tubes and extracted with 200 μL of methanol, with the addition of 5 μL of IS mixture working solution (0.500 μg/mL). After a 10 min incubation at 37 ± 1 °C, the samples were centrifuged at 20,000× *g* for 5 min at 4 °C. The supernatants were then transferred to autosampler glass vials for LC-MS/MS analysis.

### 2.5. LC-MS/MS Conditions

#### 2.5.1. Chromatographic Separation

Chromatography analyses were performed using Ultimate 3000 UHPLC Dual-Gradient Pumps (Thermo Fisher Scientific, Milan, Italy), a Hypersil Gold aQ column (2.1 mm × 50 mm, i.d. 1.9 µm, Thermo Fisher Scientific, Milan, Italy) maintained at 50 °C. Water with 0.1% *v*/*v* formic acid was used as eluent A and acetonitrile (ACN) with 0.1% *v*/*v* formic acid as eluent B, with a flow rate set at 600 µL/min. The gradient program began at 10% eluent B and ramped up to 100% eluent B over 0.7 min, which was held for 1.05 min. The column was then re-equilibrated with 10% eluent B for 1.75 min, resulting in a total run time of 3.5 min. The injection volume was 5 µL.

#### 2.5.2. Mass Spectrometric Acquisition

The system (TSQ Quantiva MS/MS) was set to operate in scheduled multiple reaction monitoring (MRM) mode. The vaporizer and capillary temperatures were maintained at 350 °C. Nitrogen was used as both the nebulizer and auxiliary gases, set to 45 and 10 arbitrary units, respectively. Argon served as the collision gas, with a pressure of 1.5 mTorr.

The specific transitions, detected using multiple reaction monitoring (MRM) were: 598.3→422.2 for ELX, 601.4→422.2 for ELX d-3, 521.3→387.1 for TEZ, 525.3→391.1 for TEZ d-4, 393.2→ 319.1 for IVA and 402.2→ 328.1 for IVA d-9,

### 2.6. Method Validation

The method has been validated according to the ICH guidelines M10 on bioanalytical methods validation [23].

#### 2.6.1. Selectivity and Specificity

For selectivity and specificity assessment, plasma samples from ten healthy volunteers who were not taking drugs and from patients on Kaftrio^®^ therapy were analyzed. For the analysis, an LLOQ (lower limit of quantitation) sample, a QC high sample and a sample spiked with an internal standard (IS) were prepared using human blank plasma. Each batch of samples was processed and analyzed using the same method. The criterion for acceptable performance was that the signal for each analyte should be less than 20% of the LLOQ, indicating the absence of interfering components.

#### 2.6.2. Carry-Over

Carry-over was assessed by analyzing triplicate blank samples after the injection of the highest calibration standard into the LC-MS/MS. The carry-over was considered negligible for a signal in the blank sample within 20% LLOQ and 5% IS.

#### 2.6.3. Matrix Effect and Extraction Recovery

Matrix effect and extraction recovery were evaluated by analyzing 6 lots of blank matrix samples from healthy donors. Matrix effect was assessed through a “T” fitting, the output of the chromatographic column was connected with a syringe, with a solution of ETI at 5 µg/mL and connected to the source. A blank matrix sample (DPS and VAMS) was injected into the column, and the solution was infused simultaneously through the syringe. The described procedure allowed us to verify if the ionization of ETI was affected by interferents present in the matrix.

Extraction recovery for ETI and IS was assessed by analyzing QC low and QC high in triplicate for each matrix (plasma, DPS and VAMS) and comparing the peak areas of all assayed analytes before extraction with the peak areas of ETI after extraction.

#### 2.6.4. Linearity

Linearity was assessed by analyzing three calibration curves on different days. The ratio of the analyte peak area to the IS was plotted against the analyte concentration for each calibration standard, with the data weighted using a 1/x factor. The calibration curves were validated in the concentration range of 0.020–12.000 mµg/mL for all analytes. The acceptance criterion for the recalculated concentrations of the calibration standards was within 15% of the theoretical value, with the exception of LLOQ, which had a tolerance of 20%.

#### 2.6.5. Precision, Accuracy and LLOQ

Intra-day precision and accuracy were assessed by analyzing the LLOQ and QC samples in quintuplicate within a single analytical run. Inter-day accuracy and precision were evaluated by testing the LLOQ and QC samples in triplicate across three separate analytical runs. Accuracy was determined as the percentage difference from the nominal value, while precision was expressed as the coefficient of variation (CV%). Accuracy was considered acceptable within 85–115% for QCs and within 80–120% for the LLOQ. Precision was deemed acceptable if the CV% was within ±15% for QCs and ±20% for the LLOQ. Additionally, the signal-to-noise ratio for the LLOQ was required to be greater than 5.

#### 2.6.6. Stability

The stability of ELX, TEZ and IVA in plasma, DPS and VAMS was assessed by analyzing three replicates of QC low and QC high after storage at +4 °C and room temperature (RT) at the following times: 3 and 7 days. The stability of stock and working solution was tested after storage at −20 °C for 4 weeks. Stability was considered acceptable if the percentage difference between the concentration measured at each sampling point and the initial concentration was within ±15%.

### 2.7. Statistical Analyses

Descriptive statistics were compiled for the entire cohort, with data presented as the mean and standard deviation for continuous variables. For categorical variables, absolute and relative frequencies were reported, while median values and ranges were calculated and documented as well. Statistical analyses were conducted using MedCalc software, ver 22.021 (MedCalc Software Ltd., Ostend, Belgium).

## 3. Results

### 3.1. Method Development

The presented method for the simultaneous quantification of ETI in different matrices (DPS and VAMS) was developed on an Ultimate 3000 UHPLC Dual-Gradient Pumps coupled to a TSQ Quantiva MS/MS in positive ionization mode. The MRM acquisition mode was used in order to set up an accurate and selective quantification method. Transitions were chosen by direct injection of pure compounds into the mass spectrometer. CID-induced fragmentation of protonated ions for all analytes produced two major daughter ions, the quantifier ion and the qualifier ion. The use of the Hypersil Gold aQ column allowed us to achieve optimal chromatographic resolution, thereby reducing interferences. The chromatograms in Supplementary Figure S1 demonstrate the excellent resolution and intensity provided by the chosen chromatographic column. The total run time was 3.5 min, with retention times of 1.79 min for ELX, 1.61 min for TEZ, and 1.71 min for IVA.

### 3.2. Method Validation

The method was validated following the ICH guidelines M10 [23] and the results obtained showed excellent precision and accuracy for the quantification of ETI in plasma, DPS and VAMS. No interfering peaks were detected under the specified LC-MS/MS conditions, and carry-over was found to be negligible. ER tests gave results within the acceptable ranges (<15% for all the analytes tested). Matrix effect evaluation revealed no significant deviations from the signal (both ion suppression and/or enhancement) at the retention times of ETI.

The lower limit of quantification (LLOQ) resulted in 0.020 µg/mL. The calibration curves for all analytes were built using quadratic equations and the ratio of the analyte peak area to the IS (analyte/IS) was plotted by using a 1/x weighting factor. The corresponding concentration was achieved across the entire concentration range, with an R^2^ value of 0.99. The back-calculated concentrations for all analytes were within ±15% of the nominal values. The mean calibration curve statistics and R^2^ for IVA, TEZ and ELX, in plasma, DPS and VAMS have been summarized in Supplementary Table S1. Supplementary Figure S2 shows the mean calibration curves for ETI in plasma (panel A), DPS (panel B) and VAMS (panel C). Results of intra- and inter-day precision and accuracy were acceptable for ETI in plasma, DPS and VAMS (Table 1). The stability results for plasma, DPS, and VAMS samples containing ETI are summarized in Table 2.

### 3.3. Analyses of Clinical Samples

A total of 21 samples derived from eight patients were analyzed. Patients were males (n = 5) and females (n = 3) with a median age of 27 years (range, 14–61 years). Eight plasma and DPS and five VAMS samples were analyzed.

Table 3 shows the concentrations obtained for plasma, DPS and VAMS. According to D’Urso and colleagues [24], the blood/plasma ratio (R) for VAMS was determined by dividing the drug concentration in VAMS by the plasma concentration. The mean R values were found to be less than 1, specifically 0.59 ± 0.07 for TEZ, 0.64 ± 0.11 for IVA, and 0.63 ± 0.09 for ELX, which is in line with the high plasma protein binding of ETI (99%). Since whole blood was used for determining ETI on VAMS, it was necessary to consider the hematocrit value (HCT) to obtain a correction factor, which allowed for the calculation of the corrected VAMS concentration values using the following formulas:

$$corrected\;VAMS\;concentration=measured\;VAMS\;concentration\;\frac{100}{100-Hct}$$

The percentage difference between plasma and DPS ETI concentrations, as well as between plasma and VAMS ETI concentrations, was calculated for each sample, with all results falling within ±20% (Table 3).

## 4. Discussion

TDM plays a pivotal role in optimizing drug efficacy and minimizing adverse drug reactions (ADRs), particularly in conditions like CF where physiological or pathological factors can significantly impact PKs [4]. Reliable methods for the quantification of ETI in plasma are a mandatory requirement to conduct PK studies. The LC-MS/MS technique is widely recognized as the gold standard for accurately quantifying drugs in biological fluids [22,25,26]. A TDM protocol for ETI could serve as a valuable tool in clinical practice to identify inadequate clinical responses resulting from insufficient drug exposure or suboptimal adherence to treatment. The timing of blood collection is critical in TDM to yield clinically relevant information [3], as improper timing can lead to inappropriate drug dosage adjustments, either resulting in subtherapeutic or toxic drug levels, compromising both patient safety and the effectiveness of the therapy. For ETI, the optimal time points for sample collection, such as maximum concentration (Cmax), Cmin, or area under the plasma concentration-time curve (AUC), remain undefined [2]. A prior study conducted by our group failed to demonstrate any significant correlation between ETI Cmin levels and the clinical response to treatment as measured by Cl-, FEV1% and BMI [10]. This suggests that pre-dose blood collection might not be the most suitable time for TDM sample collection. A strong positive linear correlation between Ctrough and AUC_0–24_ for each of the three components of ETI has been demonstrated [27], suggesting that AUC may not be the optimal pharmacokinetic parameter for ETI TDM. However, this study was conducted with only seven adult patients, and larger, more comprehensive studies are needed. Notably, a statistically significant negative association was observed between BMI and Cmax values for TEZ in patients receiving the full dose of ETI, as well as between FEV1 and ELX levels in patients on a reduced dose [11]. These findings suggest a potential role for C_max_ in TDM. Additional PK studies are needed to elucidate ETI drug exposure–clinical response and safety relationships, which remain limited. Given the necessity for multicentric PK studies to clarify any role of TDM in assessing treatment response variability, and the need to establish reference ranges and considering that monitoring ETI exposure is currently restricted to only a few centers worldwide, this study presents, for the first time, a method for the simultaneous measurement of all three components of ETI in plasma, DPS and whole blood using VAMS.

Various methods have been published for the quantification of ETI from plasma samples [10,12,13,14,15] and a method from dried blood spots (DBSs) [16]. Microsampling techniques, including DBS, DPS and VAMS, offer distinct advantages over conventional blood collection. The minimal volume required (only a few microliters) is particularly beneficial for pediatric and neonatal populations, where obtaining large volumes of blood is challenging and risky. While both VAMS and DBS offer benefits as microsampling techniques, VAMS provides additional advantages. In fact, VAMS is an advanced volumetric sampling technique that facilitates the collection of accurate whole blood volumes [19,20,21], while in DBS, the variability in blood spot size can lead to inconsistent sample volumes and thus inaccurate drug measurement. VAMS devices are designed to absorb a precise and fixed amount of blood, improving the reproducibility of results. The collection of an accurate blood volume allows us to avoid the Hct Effect: DBS analysis is influenced by the Hct level of the blood, affecting the distribution and drying pattern of the sample. VAMS minimizes this issue, leading to more reliable and consistent analyte quantification. DPS involves spotting a fixed plasma volume onto cellulose substrates, which is particularly advantageous for multicenter studies requiring external laboratory processing, given its superior stability compared to fresh plasma [18]. However, it is important to note that compared to blood collection on VAMS, DPS entails a conventional venipuncture procedure conducted by qualified healthcare personnel, followed by laboratory processing of the whole blood sample through centrifugation [18].

In the present study, to enhance method reliability and accuracy, deuterated IS is utilized for each analyte. A thorough stability study confirmed that ETI is stable in plasma, DPS and VAMS for up to one week (Table 2) facilitating convenient sample shipment. The ETI concentrations measured in plasma and DPS were found to be interchangeable, while the ETI concentrations in VAMS were lower than those in plasma. This finding aligns with expectations for drugs with a very high plasma protein binding (99%) and is consistent with Vonk et al. [16], who reported lower ETI concentrations in DBS compared to plasma. A correction factor based on the hematocrit value allowed us to calculate the equivalent plasma concentration. Cross-validation across the three matrices was not feasible, as ICH guidelines M10 specify a requirement for either a Bland–Altman test or Deming regression, both of which necessitate a minimum of 30 samples. Further studies are required to validate this VAMS-based method using capillary blood obtained from real patient finger or heel pricks and to conduct a cross-validation statistical analysis between the different matrices with an adequate sample size.

## 5. Conclusions

In this paper, we show a novel LC-MS/MS method for quantifying ETI in plasma, DPS and VAMS. This method, validated in accordance with ICH guidelines M10, is suitable for TDM and PK multicentric studies. Its application could significantly contribute to the management of ETI therapy in CF patients.

## Figures and Tables

**Table 1:** Results of intra-day and inter-day accuracy and reproducibility assays for plasma, DPS and VAMS (n, replicates; (n) = 5). The quality control concentrations were, respectively, 0.020, 0.060, 0.400 and 10.000 µg/mL for LLOQ, QC low, QC medium and QC high. (SD, Standard deviation; CV%, coefficient of variation percentage).

**IVACAFTOR**								
**Plasma**								
	**INTRA-DAY**				**INTER-DAY**			
	Conc. mean (µg/mL)	SD	CV%	Accuracy%	Conc. mean (µg/mL)	SD	CV%	Accuracy%
LLOQ	0.023	2.48 × 10^−3^	11%	115%	0.023	1.91 × 10^−3^	8%	115%
QC low	0.055	2.74 × 10^−3^	5%	91%	0.055	1.93 × 10^−3^	4%	91%
QC medium	0.384	1.26 × 10^−2^	3%	96%	0.372	3.08 × 10^−2^	8%	93%
QC high	9.400	7.75 × 10^−1^	8%	94%	9.800	3.61 × 10^−1^	4%	98%
DPS								
	INTRA-DAY				INTER-DAY			
	Conc. mean (µg/mL)	SD	CV%	Accuracy%	Conc. mean (µg/mL)	SD	CV%	Accuracy%
LLOQ	0.021	8.31 × 10^−4^	4%	115%	0.023	1.51 × 10^−3^	7%	114%
QC low	0.065	3.79 × 10^−3^	6%	108%	0.065	1.96 × 10^−3^	3%	109%
QC medium	0.436	2.28 × 10^−2^	5%	109%	0.436	2.93 × 10^−2^	7%	109%
QC high	9.600	3.87 × 10^−1^	4%	96%	9.400	7.28 × 10^−1^	8%	94%
VAMS								
	INTRA-DAY				INTER-DAY			
	Conc. mean (µg/mL)	SD	CV%	Accuracy%	Conc. mean (µg/mL)	SD	CV%	Accuracy%
LLOQ	0.021	2.10 × 10^−3^	10%	110%	0.022	1.54 × 10^−3^	7%	114%
QC low	0.053	4.22 × 10^−3^	7%	107%	0.059	6.18 × 10^−3^	10%	99%
QC medium	0.420	4.50 × 10^−2^	11%	105%	0.420	2.52 × 10^−2^	6%	105%
QC high	9.520	4.74 × 10^−1^	5%	95%	9.120	7.32 × 10^−1^	8%	91%
**TEZACAFTOR**								
**Plasma**								
	**INTRA-DAY**				**INTER-DAY**			
	Conc. mean (µg/mL)	SD	CV%	Accuracy%	Conc. mean (µg/mL)	SD	CV%	Accuracy%
LLOQ	0.021	1.60 × 10^−3^	8%	106%	0.022	2.14 × 10^−3^	10%	112%
QC low	0.053	3.44 × 10^−3^	6%	88%	0.052	1.57 × 10^−3^	3%	87%
QC medium	0.364	1.23 × 10^−2^	3%	91%	0.364	2.47 × 10^−2^	7%	91%
QC high	10.520	7.83 × 10^−1^	7%	105%	10.830	9.96 × 10^−1^	9%	108%
DPS								
	INTRA-DAY				INTER-DAY			
	Conc. mean (µg/mL)	SD	CV%	Accuracy%	Conc. mean (µg/mL)	SD	CV%	Accuracy%
LLOQ	0.022	1.66 × 10^−3^	8%	110%	0.022	2.92 × 10^−3^	13%	112%
QC low	0.064	5.26 × 10^−3^	8%	7%	0.064	5.14 × 10^−3^	8%	106%
QC medium	0.448	2.68 × 10^−2^	6%	12%	0.440	1.97 × 10^−2^	4%	110%
QC high	8.490	4.85 × 10^−^	6%	85%	9.370	6.55 × 10^−1^	7%	94%
VAMS								
	INTRA-DAY				INTER-DAY			
	Conc. mean (µg/mL)	SD	CV%	Accuracy%	Conc. mean (µg/mL)	SD	CV%	Accuracy%
LLOQ	0.023	1.18 × 10^−3^	5%	15%	0.023	1.43 × 10^−3^	6%	115%
QC low	0.059	2.83 × 10^−3^	5%	−7%	0.058	2.76 × 10^−3^	5%	97%
QC medium	0.408	3.69 × 10^−3^	1%	2%	0.412	2.35 × 10^−2^	6%	103%
QC high	9.420	7.97 × 10^−1^	8%	−6%	9.800	5.76 × 10^−1^	6%	98%
**ELEXACAFTOR**								
**Plasma**								
	**INTRA-DAY**				**INTER-DAY**			
	Conc. mean (µg/mL)	SD	CV%	Accuracy%	Conc. mean (µg/mL)	SD	CV%	Accuracy%
LLOQ	0.021	1.07 × 10^−3^	5%	105%	0.022	2.30 × 10^−3^	10%	110%
QC low	0.058	4.80 × 10^−3^	8%	98%	0.057	2.87 × 10^−3^	5%	95%
QC medium	0.404	2.15 × 10^−2^	5%	101%	0.384	3.25 × 10^−2^	8%	96%
QC high	9.910	6.13 × 10^−1^	6%	99%	9.950	4.66 × 10^−1^	5%	100%
DPS								
	INTRA-DAY				INTER-DAY			
	Conc. mean (µg/mL)	SD	CV%	Accuracy%	Conc. mean (µg/mL)	SD	CV%	Accuracy%
LLOQ	0.023	1.41 × 10^−3^	6%	13%	0.022	1.18 × 10^−3^	5%	112%
QC low	0.065	2.83 × 10^−3^	4%	9%	0.065	2.72 × 10^−3^	4%	108%
QC medium	0.480	2.62 × 10^−2^	5%	9%	0.432	2.04 × 10^−2^	5%	108%
QC high	10.860	8.66 × 10^−1^	8%	8%	9.520	6.11 × 10^−1^	6%	95%
VAMS								
	INTRA-DAY				INTER-DAY			
	Conc. mean (µg/mL)	SD	CV%	Accuracy%	Conc. mean (µg/mL)	SD	CV%	Accuracy%
LLOQ	0.023	4.90 × 10^−4^	2%	15%	0.023	5.21 × 10^−4^	2%	115%
QC low	0.065	3.19 × 10^−3^	5%	9%	0.064	5.78 × 10^−3^	9%	106%
QC medium	0.436	2.02 × 10^−2^	5%	9%	0.420	3.28 × 10^−2^	8%	105%
QC high	9.700	5.55 × 10^−1^	6%	−3%	9.820	5.52 × 10^−1^	6%	98%

CV%, percentage coefficient of variation; DPS, dried plasma spot; LLOQ, lower limit of quantification; QC, quality control; SD, Standard deviation; VAMS, Volumetric Absorptive Microsampling.

**Table 2:** Stability of IVA, TEZ and ELX measured on plasma, DPS and VAMS. Results are expressed as the accuracy and CV percentage (CV%) (CV% is the coefficient of variation percentage).

	**IVACAFTOR**					
	**7 days**					
	**+4 °C**			**+25 °C (RT)**		
	Plasma	DPS	VAMS	Plasma	DPS	VAMS
QC low	105% (2%)	103% (7%)	98% (6%)	95% (4%)	93% (5%)	92% (5%)
QC high	109% (2%)	105% (5%)	91% (3%)	106% (2%)	92% (6%)	94% (2%)
	3 days					
	+4 °C			+25 °C (RT)		
	Plasma	DPS	VAMS	Plasma	DPS	VAMS
QC low	102% (5%)	95% (2%)	98% (6%)	96% (2%)	94% (3%)	93% (6%)
QC high	109% (4%)	107% (3%)	97% (5%)	105% (3%)	104% (6%)	92% (4%)
	TEZACAFTOR					
	7 days					
	+4 °C			+25 °C (RT)		
	Plasma	DPS	VAMS	Plasma	DPS	VAMS
QC low	104% (2%)	90% (6%)	92% (5%)	93% (2%)	96% (5%)	95% (4%)
QC high	105% (3%)	102% (4%)	95% (4%)	109% (4%)	99% (6%)	99% (5%)
	3 days					
	+4 °C			+25 °C (RT)		
	Plasma	DPS	VAMS	Plasma	DPS	VAMS
QC low	99% (3%)	94% (5%)	92% (5%)	94% (2%)	91% (5%)	93% (3%)
QC high	109% (5%)	101% (4%)	94% (2%)	106% (1%)	99% (3%)	98% (6%)
	ELEXACAFTOR					
	7 days					
	+4 °C			+25 °C (RT)		
	Plasma	DPS	VAMS	Plasma	DPS	VAMS
QC low	100% (4%)	98% (6%)	93% (2%)	99% (4%)	91% (2%)	98% (4%)
QC high	101% (3%)	99% (7%)	97% (5%)	108% (2%)	94% (3%)	92% (3%)
	3 days					
	+4 °C			+25 °C (RT)		
	Plasma	DPS	VAMS	Plasma	DPS	VAMS
QC low	103% (2%)	94% (2%)	98% (5%)	98% (2%)	99% (2%)	97% (5%)
QC high	103% (3%)	106% (2%)	99% (4%)	108% (3%)	105% (3%)	98% (4%)

**Table 3:** Results of ETI concentrations (expressed in µg/mL) in plasma, VAMS and DPS. * Concentration value multiplied by the conversion factor.

**ELEXACAFTOR**							
**Patient**	**Plasma concentration (** **µg/mL)**	**DPS concentration (** **µg/mL)**	**VAMS concentration measured (** **µg/mL)**	**VAMS concentration corrected (** µg/mL) *	**% difference plasma vs. DPS**	**% difference plasma vs. VAMS**	**Blood-to-Plasma ratio (R)**
1	3.559	3.666	-	-	3%	-	-
2	4.509	4.248	-	-	−6%	-	-
3	6.479	5.961	-	-	−8%	-	-
4	8.343	7.387	4.462	8.099	−11%	3%	0.53
5	2.897	2.947	1.698	2.968	2%	−2%	0.59
6	3.477	3.161	1.973	3.518	−9%	−1%	0.57
7	8.654	7.965	6.052	9.545	−8%	−10%	0.70
8	3.572	3.535	2.677	4.208	−1%	−18%	0.75
**TEZACAFTOR**							
**Patient**	**Plasma concentration (** **µg/mL)**	**DPS concentration (** **µg/mL)**	**VAMS concentration measured (** **µg/mL)**	**VAMS concentration corrected (** µg/mL) *	**% difference plasma vs. DPS**	**% difference plasma vs. VAMS**	**Blood-to-Plasma ratio (R)**
1	2.919	3.100	-	-	6%	-	-
2	1.688	1.621	-	-	-4%	-	-
3	3.483	3.403	-	-	-2%	-	-
4	3.267	2.895	1.721	3.124	−12%	4%	0.53
5	1.159	1.148	0.601	1.050	−1%	9%	0.52
6	1.599	1.435	0.846	1.508	−11%	6%	0.53
7	2.631	2.481	1.801	2.847	−6%	−8%	0.69
8	1.163	1.040	0.736	1.235	−10%	−6%	0.63
**IVACAFTOR**							
**Patient**	**Plasma concentration (** **µg/mL)**	**DPS concentration (** **µg/mL)**	**VAMS concentration measured (** **µg/mL)**	**VAMS concentration corrected (** µg/mL) *	**% difference plasma vs. DPS**	**% difference plasma vs. VAMS**	**Blood-to-Plasma ratio (R)**
1	0.757	0.804	-	-	5%	-	-
2	0.758	0.793	-	-	4%	-	-
3	1.552	1.616	-	-	5%	-	-
4	0.999	0.883	0.507	0.919	−12%	8%	0.51
5	0.357	0.384	0.201	0.351	6%	2%	0.56
6	0.130	0.127	0.072	0.128	0%	2%	0.55
7	1.234	1.179	0.860	1.356	−4%	−10%	0.70
8	0.441	0.436	0.317	0.498	0%	−20%	0.72

## Data Availability

The original contributions presented in this study are included in the article/Supplementary Materials. Further inquiries can be directed to the corresponding author.

## Supplementary Materials

The following supporting information can be downloaded at: https://www.mdpi.com/article/10.3390/pharmaceutics17020200/s1. Figure S1: Chromatograms obtained from the analysis of ETI in plasma (panel A), DPS (panel B) and VAMS (panel C). RT is retention time. Figure S2. Mean calibration curves (8 point calibration curve) of IVA, TEZ and ELX ranging from 0.02 to 12.00 mg/L in plasma (panel A), in DPS (panel B) and in VAMS (panel C). Table S1: The equation of mean calibration curve statistics and R^2^ for IVA, TEZ and ELX, in plasma, DPS and VAMS.
