# Supplementary material for: A Novel LC-MS/MS Method for the Measurement of Elexacaftor, Tezacaftor and Ivacaftor in Plasma, Dried Plasma Spot (DPS) and Whole Blood in Volumetric Absorptive Microsampling (VAMS) Devices
Source: Pharmaceutics. 2025 Feb 6;17(2):200. doi: 10.3390/pharmaceutics17020200 (PMC11859332; doi:10.3390/pharmaceutics17020200)
Supplement: Supplementary file 1 [file pharmaceutics-17-00200-s001.zip › pharmaceutics-3417159-supplementary.pdf]

SUPPLEMENTARY MATIRIALS

Figure S1: Chromatograms obtained from the analysis of ETI in plasma (panel A), DPS (panel B) and VAMS (panel C). RT is retention time.

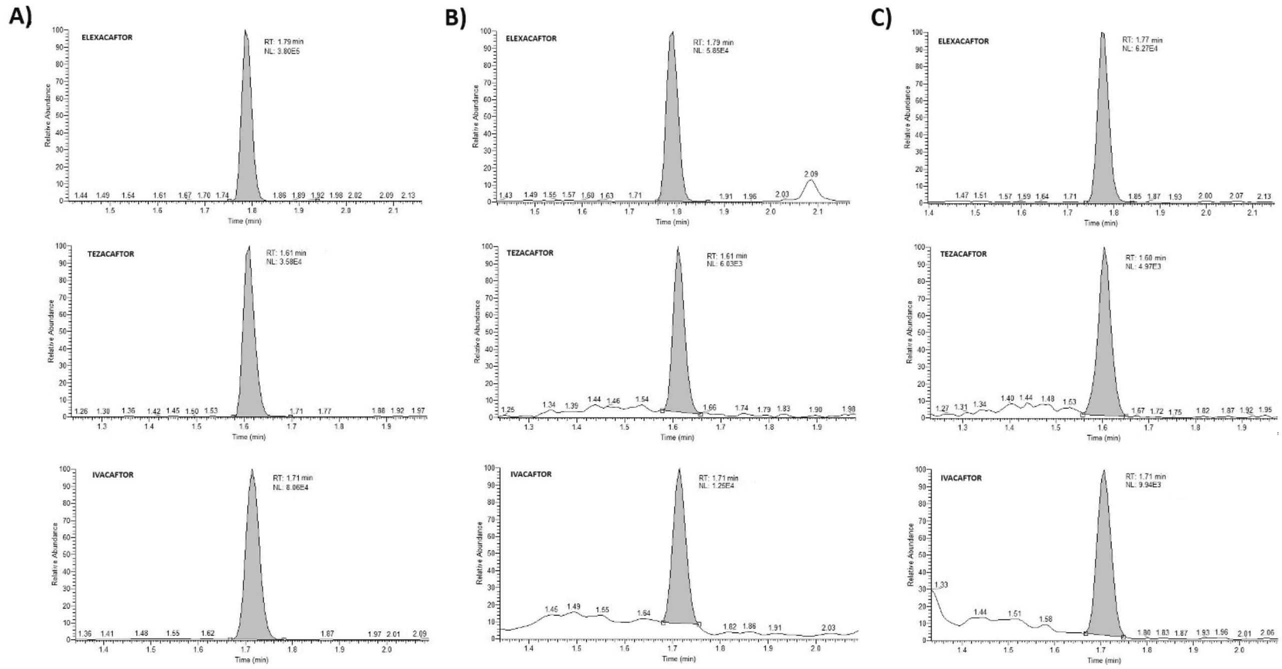

Figure S2. Mean calibration curves (8 point calibration curve) of IVA, TEZ and ELX ranging from 0.02 to 12.00 mg/L in plasma (panel A), in DPS (panel B) and in VAMS (panel C).

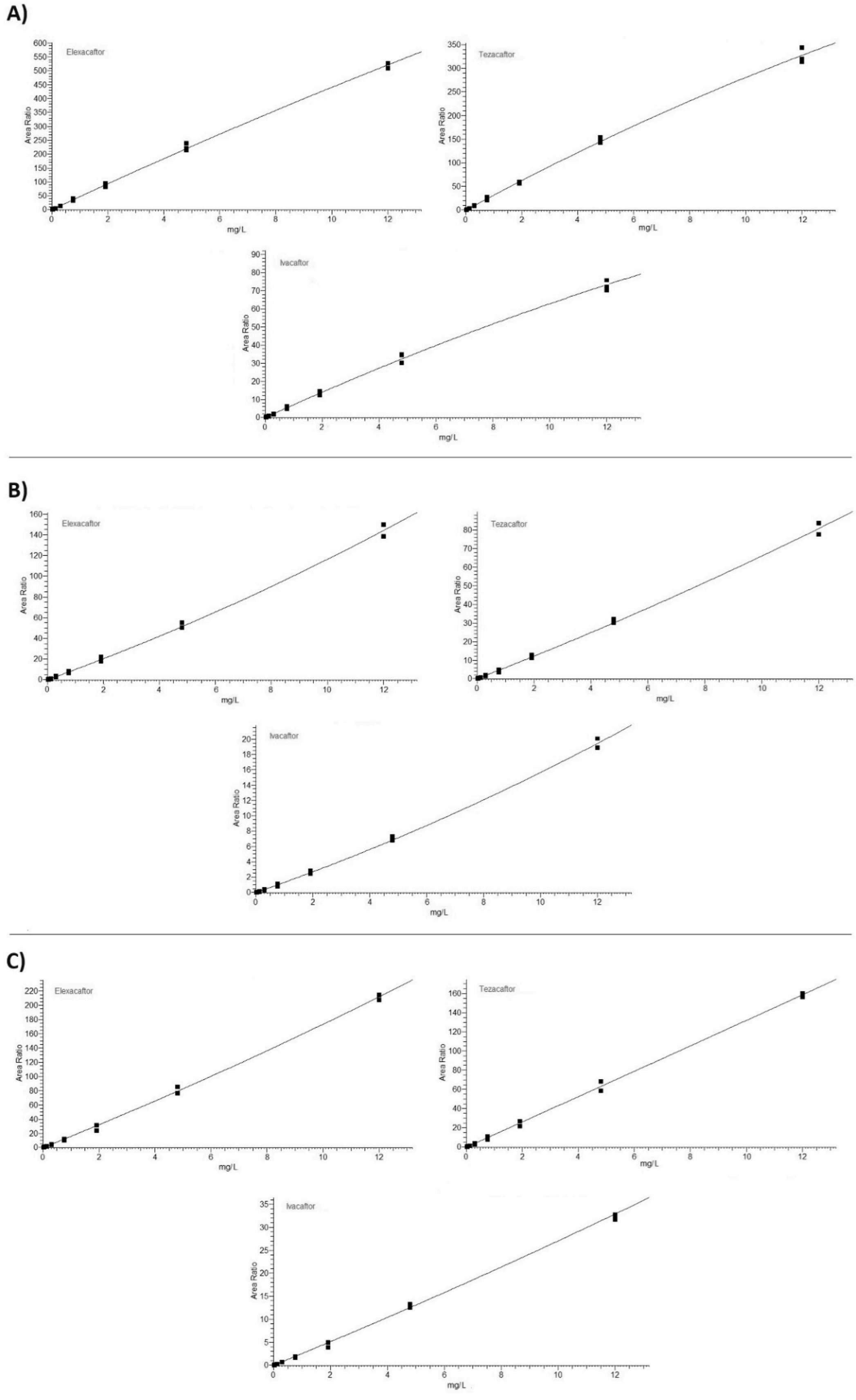

Table S1. The equation of mean calibration curve statistics and R^2 for IVA, TEZ and ELX, in plasma, DPS and VAMS.

PLASMA			
	IVA	TEZ	ELX
Equation	$Y = -6.6 \times 10^{-2} + 7.1 \times 10^{-3} X - 8.7 \times 10^{-8} \times X^2$	$Y = -2.0 \times 10^{-1} + 3.2 \times 10^{-2} \times X - 4.0 \times 10^{-7} \times X^2$	$Y = -4.1 \times 10^{-1} + 4.7 \times 10^{-2} X – 3.2 \times 10^{-7} \times X^2$
R^2	0.995	0.998	0.998
DPS			
	IVA	TEZ	ELX
Equation	$Y = -9.8 \times 10^{-3} + 1.3 \times 10^{-3} X + 2.7 \times 10^{-8} \times X^2$	$Y = -3.8 \times 10^{-2} + 5.9 \times 10^{-3} \times X + 6.6 \times 10^{-8} \times X^2$	Y = -3.7 x 10-2 + 9.8 x 10-3 X + $1.9 \times 10^{-7} \times X^2$
R^2	0.996	0.997	0.996
VAMS			
	IVA	TEZ	ELX
Equation	$Y = -4.7 \times 10^{-3} + 2.5 \times 10^{-3} X + 1.8 \times 10^{-8} \times X^2$	$Y = -1.5 \times 10^{-1} + 1.3 \times 10^{-2} \times X + 8.6 \times 10^{-9} \times X^2$	$Y = -7.7 \times 10^{-2} + 1.6 \times 10^{-2} X + 1.6 \times 10^{-7} \times X^2$
R^2	0.998	0.996	0.998
